# The Impact of *κ*‐Carrageenan on the Textural, Microstructural, and Molecular Properties of Heat‐Induced Egg White Protein Gel

**DOI:** 10.1002/fsn3.70541

**Published:** 2025-08-03

**Authors:** Yuping Wei, Shaomin Lin, Weicong Lin, Ying Nie, Xianghui Zou, Yuzhong Zheng, Biansheng Li, Yuwei He, Yisheng Huang, Yongping Huang

**Affiliations:** ^1^ School of Food Engineering and Biotechnology Hanshan Normal University Chaozho China; ^2^ Guangdong Provincial Key Laboratory of Functional Substances in Medicinal Edible Resources and Healthcare Products Chaozhou China; ^3^ Artemisinin Research Center Guangzhou University of Chinese Medicine Guangzhou China; ^4^ College of Food Science and Engineering South China University of Technology Guangzhou China

**Keywords:** *κ*‐carrageenan, egg white protein, gel properties, heat‐induced gelation

## Abstract

In this study, we investigated the ability of *κ*‐carrageenan to reinforce the heat‐induced EWP/Car gel system. A series of composite gels containing 0%–1.0% *κ*‐carrageenan was prepared and characterized. The results showed that moderate *κ*‐carrageenan addition (particularly 0.6%) significantly improved gel hardness, elasticity, and water‐holding capacity. Rheological and microstructural analyses confirmed that *κ*‐carrageenan promoted the formation of a denser, more homogeneous network, as evidenced by lower tan*δ* values and uniform fluorescence and pore distribution in CLSM and SEM images. FTIR and SDS‐PAGE results indicated that these enhancements were mediated by noncovalent interactions without altering protein secondary structures. Moreover, the reduction in free sulfhydryl groups and increased disulfide bond formation revealed a strengthened protein matrix. These findings provide promising insights for the rational design and application of EWP‐based systems in the production and processing of egg‐based food products.

## Graphical abstract

Based on the graphical abstract, this study demonstrates that increasing *κ*‐carrageenan concentration enhances the gelation properties of egg white protein gels. The addition of *κ*‐carrageenan improves gel hardness, elasticity, and water‐holding capacity while forming a denser network structure, as shown by CLSM and SEM analysis. Rheological and T2 measurements confirm the structural stability and viscoelastic behavior. These findings highlight the potential applications of EWP/Car gels in protein‐rich food products such as snacks, cold dishes, and 3D‐printed nutrition gels.

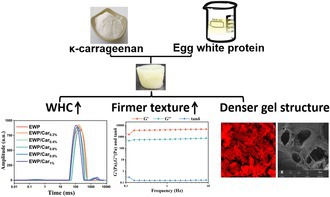

## 1 Introduction

Egg white protein (EWP) is rich in proteins and exhibits a variety of functional properties, such as foaming, emulsification, and gelation, making it an essential resource in food processing. Among these, gelation is one of the most commonly utilized functional properties in industrial production. Heat induced is a traditional method for preparing egg white protein gels, during which gelation involves conformational changes or partial denaturation of the protein, typically involving folding and dissociation (Liu et al. 2023). Key components in egg white form the foundational basis for gelation (Souza and Garcia‐Rojas 2015). Changes in hydrogen bonding, van der Waals forces, and hydrophobic interactions promote gel formation (He et al. 2023; Xiong et al. 2024).

Heating, as the most common processing method in food production, induces structural changes in proteins, thereby affecting their functional properties. Protein aggregation leads to a reduction in solubility, which significantly impacts critical functionalities such as foaming, emulsification, and gelation (Claude et al. 2017). EWP exhibits excellent heat‐induced gelation capability; however, the resulting gel texture often lacks sufficient strength and elasticity, tending to become loose and brittle. These shortcomings limit its applicability in the food industry. Many studies have shown that polysaccharides alter the intermolecular and intramolecular interactions within protein‐polysaccharide complexes, thereby influencing the gelation properties of proteins (Dickinson and Pawlowsky 1997); (Lv et al. 2022; Wang et al. 2020). The interactions between proteins and polysaccharides can lead to either segregative or associative effects, making the study of these interactions crucial for developing innovative food products with tailored functions and properties (Chantanuson et al. 2024; Zhang, Xia, and Peng 2024).

*κ*‐Carrageenan, a polysaccharide extracted from red seaweed, is widely used in the food industry due to its thickening, stabilizing, and gelling properties (Zhou et al. 2023). As a widely approved food additive, *κ*‐carrageenan enhances the water‐holding capacity of protein‐based systems at relatively low concentrations, thereby extending the shelf life of food products and improving their overall texture. For example, the addition of only 0.02% carrageenan in cream effectively prevents fat droplet flocculation during storage, thus prolonging its shelf life (Xu, Gao, et al. 2023). Similarly, the incorporation of just 0.5% *κ*‐carrageenan into a solution containing 7.5% heat‐induced pea protein aggregates results in the formation of more ordered protein gels, significantly improving the mechanical strength of the heat‐induced gel (Cai et al. 2021). In another study, the critical role of varying *λ*‐carrageenan concentrations (0.25%, 0.375%, 0.5%, 0.625%, and 0.75%, w/w) in the gel structure of acid‐induced soy protein isolate/λ‐carrageenan (SPI/Car) systems was investigated. The results indicated that λ‐carrageenan formed a network structure with SPI particles through electrostatic interactions and hydrogen bonding. As the concentration of λ‐carrageenan increased, hydrogen bonding and electrostatic interactions were enhanced, leading to improved water‐holding capacity (WHC) and gel hardness of the SPI/Car gels (Dongling et al. 2023).

EWP inherently possesses good heat‐induced gelation capability; however, its gel texture often lacks sufficient strength and elasticity, making it prone to being loose and brittle. The addition of *κ*‐carrageenan can synergistically improve the heat‐induced gelation properties of EWP, resulting in a more elastic and flexible gel network. Moreover, *κ*‐carrageenan enhances the water‐holding capacity of protein‐based systems, thereby extending the shelf life of food products and improving their overall texture. At present, *κ*‐carrageenan is widely utilized as an important food additive in jelly products, soft candies, meat products, and bakery products, playing a critical role in food processing. However, current research has primarily focused on its interactions with plant proteins and dairy proteins, whereas the interaction mechanisms between *κ*‐carrageenan and avian egg proteins remain unclear. In particular, a systematic investigation is needed to elucidate the dose‐dependent effects of *κ*‐carrageenan on the microstructure and molecular driving forces of EWP gels.

This study aimed to address the limitations of EWP gels, such as poor elasticity and brittleness, by investigating the synergistic effects of *κ*‐carrageenan on their textural, structural, and functional properties. We examined the performance changes in the EWP/Car gels, including their textural properties, water‐holding capacity (WHC), moisture distribution, intermolecular forces, infrared characteristics, microrheological performance, and free sulfhydryl group content, alongside the gel's appearance and microstructural outcomes. This comprehensive analysis elucidates the gelation behavior of the composite EWP/Car protein, providing a theoretical foundation for the application of *κ*‐carrageenan and EWP in composite food gels. By elucidating the structure–function relationship, the findings are expected to provide a theoretical foundation and practical guidance for improving the formulation and processing of egg‐based gel products, including high‐moisture protein snacks, refrigerated egg dishes, and customizable 3D‐printed nutritional gels.

## 2 Materials and Methods

### 2.1 Materials

Pasteurized liquid hen's egg white was supplied from WuQiong Food Co. Ltd. (GuangDong, China), which produces liquid egg white daily without any processing aids or additives. It was stored at temperatures ranging from 2°C to 8°C until utilized. The protein content of EWP was determined to be 11.0% using the Bradford protein assay. *κ*‐Carrageenan (Purity: 99%, Mr.: 788.7, average molecular weight: 600–800 kDa, and sulfate content: approximately 25%–30%) was purchased from GreenFresh Foodstuff Co. Ltd. (Fujian, China) and was used without further purification. PAGE Gel Fast Preparation Kit (G2004), 5 × loading buffer (G2075), and protein Marker (G2058) were purchased from Servicebio (Wuhan, China). Other chemicals used in this study were analytical grade reagents (Sinopharm Chemical Reagent Co. Ltd., Shanghai, China).

### 2.2 Preparation of Sample

Heat‐induced EWP/Car gels were prepared based on previous reports with some modifications (Dongling et al. 2023). Briefly, weighed *κ*‐carrageenan (0, 0.2, 0.4, 0.6, 0.8 and 1 g) and EWP (60 g) were added to a beaker containing 40 mL of ultrapure water, which was subsequently stirred with an electric stirrer (LC‐ES‐60, Lichen Instrument Technology Co., Hunan, China) at 700 rpm for 10 min and heated at 90°C for 30 min to make a gel. The prepared gel was cooled to room temperature and stored at 4°C for 24 h. The samples were labeled as EWP, EWP/Car_0.2%_, EWP/Car_0.4%_, EWP/Car_0_._6%_, EWP/Car_0_._8%_, and EWP/Car_1%_.

### 2.3 Determination of the Color Difference

A nh310 colorimeter (3NH Technology Co. Ltd., Shenzhen, China) was used for color value measurement, including: brightness (L*), redness (a*), and yellowness (b*). The final result for each sample is the average of three parallel experiments. The total color difference (ΔE) of gel samples is calculated using the following formula:

$$\Delta E=\sqrt{{\left(L_{0}*-L*\right)}^{2}+{\left(a_{0}*-a*\right)}^{2}+{\left(b_{0}*-b*\right)}^{2}}$$

where L_0_*, a_0_*, and b_0_* represent the color parameters of the gel.

### 2.4 Texture Profile Analysis (TPA)

The texture of the samples was determined using a texture analyzer (TFC‐TMS‐PRO, USA). The EWP/Gar gels were cut into cylindrical shapes (15 mm in height, 30 mm in diameter). The testing was performed in a texture analyzer (TFC‐TMS‐PRO, USA) equipped with probe P50: a testing speed of 60 g/min, a trigger force of 0.038 N, and a load cell capacity of 25 N.

### 2.5 Water‐Holding Capacity (WHC)

The water‐holding capacity (WHC) was calculated as the weight ratio of the gel after centrifugation (4°C, 4000 r/min for 15 min) to the weight ratio of the gel before centrifugation (Jiang et al. 2024).

### 2.6 Determination of Low‐Field Nuclear Magnetic Resonance Spectroscopy (LF‐NMR)

NMR relaxation measurements of heat‐induced gels were performed using low‐field NMR. Take a 5 g egg white gel and place it in a nuclear magnetic resonance (NMR) tube to measure its transverse relaxation time (T_2_). The proton resonance frequency is set at 12 MHz, and the testing temperature is set at 25°C. The transverse relaxation time (T_2_) is determined using the Carr‐Purcell‐Meiboom‐Gill (CPMG) sequence. The spectral width (SW) is set to 200 kHz, the receiver gain (RG) is set to 20 dB, and the number of scans (NS) is set to 4 (Jin et al. 2021).

### 2.7 Measurements of Rheological Properties

Rheological measurements were performed using a rheometer (Haake Mars 40, Thermo Fisher Scientific, Germany). Samples were placed between a parallel plate geometry with a 1 mm gap. Frequency sweep tests were conducted over a range of 0.1–100 Hz at 25°C to ensure measurements within the linear viscoelastic region.

### 2.8 Measurements of Micro‐Rheology by Diffuse Wave Spectroscopy

The rheological properties of the EWP/Gar gels were measured using the Rheolaser Master optical micro rheometer (Formulaction Instruments, Toulouse, France). The following parameters were set: sample volume of 20 mL and temperature of 25°C. The transport mean free path (L), elasticity index (EI), and solid–liquid balance (SLB) were obtained for each sample (Zhao et al. 2023).

### 2.9 Determination of Microstructure

Confocal laser‐scanning microscope (CLSM) images of the heat‐induced gels were acquired using a TCS SP8 confocal scanning laser microscope (LEICA Microsystems Inc., Heidelberg, Baden‐Wurttemberg, Germany) with an excitation wavelength of 547 nm (Dongling et al. 2023). Staining was performed by adding 30 μL of 0.01% Rhodamine B to the thin section of the sample, followed by incubation at room temperature in the dark for 15 min. Subsequently, 100 μL of PBS was added from one side, and excess Rhodamine B was removed by blotting with absorbent paper. The sample was then covered with a coverslip, inverted, and imaged using the CLSM. At least six images were captured at a magnification of 200× for each sample (Tian et al. 2024).

The gel samples were freeze‐dried under vacuum, coated with a thin layer of gold using a sputter coater, and subsequently observed for microstructural morphology using a scanning electron microscope (SEM) (ZEISS Sigma 360, Germany).

### 2.10 Fourier Transform Infrared (FTIR) Spectroscopy

The freeze‐dried EWP/Gar gel was powdered and ground with potassium bromide (KBr), and then pressed into pellets. Following the method described in the previously reported method (Shi et al. 2023), the samples were scanned using an FTIR spectrophotometer (Nicolet iS 10, ThermoFisher Scientific, USA) in the range of 4000–400 cm^−1^, with a resolution of 4 cm^−1^. The second derivative of the protein amide I region (ranging from 1600 to 1700 cm^−1^) was processed by using OMNIC 9.0 data processing software.

### 2.11 Determination of the Free Sulfhydryl Contents

The gel sample (0.5 g) was taken and added to 10 mL Tris–HCl buffer solution (200 mM, pH 8.8), homogenized, and centrifuged at 8000 r/min for 10 min to collect the supernatant. The protein concentration of the supernatant was determined by the Bradford method. Subsequently, 2 mL of supernatant was taken and mixed with 2 mL of Tris‐Gly buffer (0.086 M Tris, 0.09 M glycine, 0.04 M EDTA, pH 8.0) for the determination of free SH group content. 40 μL of Ellman's reagent was added to the mixed solution. The reaction was carried out at 40°C for 40 min to detect the absorbance value of the sample solution at 412 nm. The free SH content was calculated as follows (Xin et al. 2023).

$$\mathrm{SH}\left(\mathrm{\mu M}/g\;\text{protein}\right)=A_{412}\times 75.53\times D/C$$

where *A*_412_ refers to the wavelength of 412 nm, 75.53 is obtained by unit conversion (10^6^/1.36 × 10^4^ M^−1^ cm^−1^), *D* is the dilution factor (15.1), and *C* is the protein concentration of the supernatant (mg/mL).

### 2.12 SDS‐Polyacrylamide Gel Electrophoresis (SDS‐PAGE)

The EWP/Gar gel was dissolved in distilled water (protein concentration at 1 mg/mL), followed by centrifugation, and the supernatant was collected. A 40 μL aliquot of the supernatant was mixed with 10 μL of 5× loading buffer. The mixture was incubated at 95°C for 5 min, then cooled in an ice bath before electrophoresis was performed. For electrophoresis, the samples were loaded onto a 12% SDS‐PAGE gel using PAGE Gel Fast Preparation Kit. Load 10 μL samples and 5 μL protein marker onto the gel. Electrophoresis was conducted using a vertical gel electrophoresis system (Bio‐Rad Co. Ltd., USA). The voltage was set to 80 V for the stacking gel and increased to 120 V when the sample reached the separating gel. Subsequently, staining was performed with Coomassie Brilliant Blue R‐250 solution for 2 h, followed by destaining with a solution of 10% methanol and 10% acetic acid until clear protein bands were visible. The gel was examined using an imaging system (Bio‐Rad Co. Ltd., USA) (Xin et al. 2023).

### 2.13 Intermolecular Force Analysis

Based on the study by Jin et al. (Jin et al. 2021): gel samples (1 g) were mixed with four denaturing solutions (10 mL) as follows: S0 (0.05 M NaCl), S1 (0.6 M NaCl), S2 (1.5 M urea + 0.6 M NaCl), S3 (8 M urea + 0.6 M NaCl), and S4 (0.5 M β‐mercaptoethanol + 0.6 M NaCl + 8 M urea). After vortexing for 5 min, the samples were shaken in a water bath at 180 r/min for 1 h. Then, centrifuged at 10,000 r/min for 30 min at 4°C to preserve the supernatant. Finally, the protein content of the supernatant was determined using the Bradford assay. The protein content of S1–S0, S2–S1, S3–S2, and S4–S3 represents the content of ionic bonds, hydrogen bonds, hydrophobic interactions, and disulfide bonds, respectively.

### 2.14 Statistical Analysis

Data were statistically analyzed using SPSS 25.0 (IBM, Chicago, USA) and Origin 2021 (Originlab Corporation, Northampton, MA, USA). *p* < 0.05 was considered a statistically significant difference.

## 3 Results and Discussion

### 3.1 Morphological Characteristics

**FIGURE 1:**
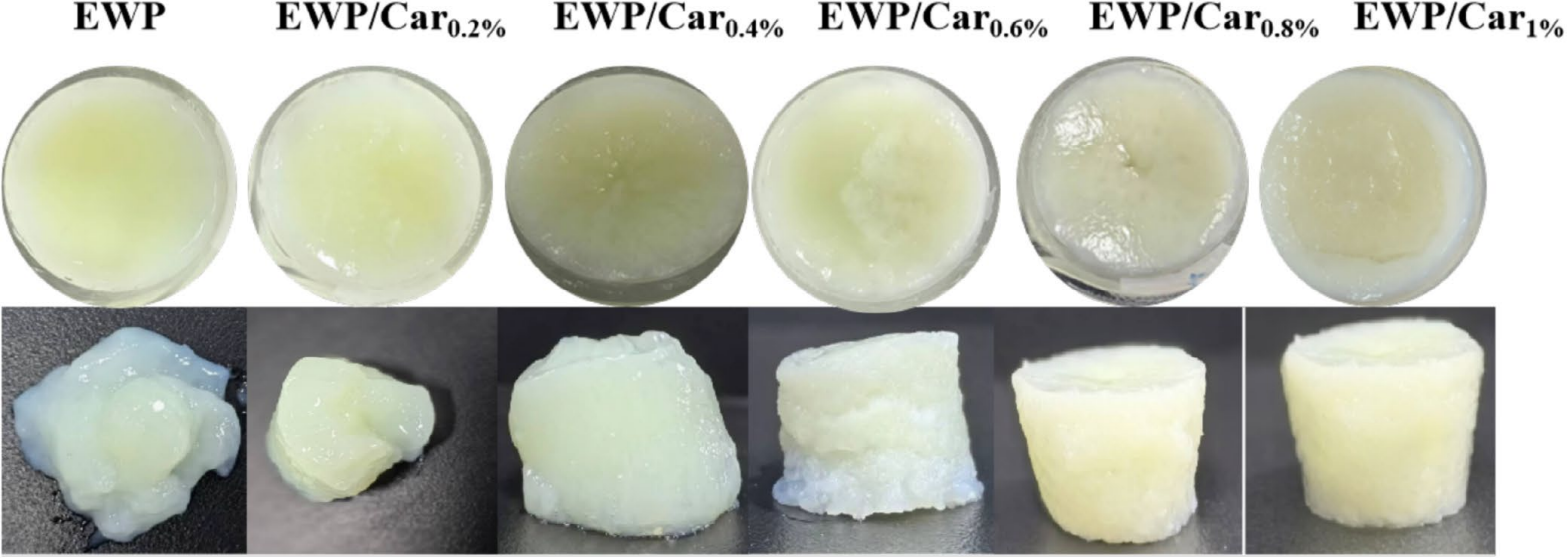
Optical photos of heat‐induced EWP gels with different concentrations of *κ*‐carrageenan.

Figure 1 visually presents the appearance of EWP and EWP/*κ*‐carrageenan (EWP/Car) gels, highlighting subtle differences. According to Table 1, the EWP gel exhibits the lowest values for a* and b*. With increasing *κ*‐carrageenan concentration, there is a notable rise in the values of a*, b*, and ΔE in the gels, with these differences being statistically significant (*p* < 0.05). The differences in L* among the four gel groups are not statistically significant. While the addition of *κ*‐carrageenan influences the color of the composite gels, the overall color difference remains marginal (*ΔE* < 2.3).

**TABLE 1:** Color difference values of gels.

Sample	L*	A*	B*	ΔE
EWP	39.44 ± 1.23^a^	−2.70 ± 0.1^c^	−0.36 ± 0.03^e^	
EWP/Car_0.2%_	39.78 ± 2.05^a^	−2.55 ± 0.32^c^	−0.30 ± 0.011^e^	0.88 ± 0.044^d^
EWP/Car_0_._4%_	40.18 ± 2.31^a^	−2.00 ± 0.18^b^	−0.13 ± 0.015^d^	0.96 ± 0.073^c^
EWP/Car_0.6%_	39.82 ± 1.98^a^	−1.31 ± 0.11^a^	0.31 ± 0.022^c^	1.59 ± 0.125^b^
EWP/Car_0.8%_	39.59 ± 4.26^a^	−1.17 ± 0.093^a^	1.04 ± 0.087^b^	2.08 ± 0.281^a^
EWP/Car_1%_	39.45 ± 3.88^a^	−1.22 ± 0.14^a^	0.79 ± 0.22^a^	2.01 ± 0.148^a^

*Note:* Values are expressed as mean ± standard deviation of triplicate samples. Values with different lowercase superscripts within a column differ significantly (*p* < 0.05).

Figure 1 shows that the EWP gel is a structureless paste. When the *κ*‐carrageenan concentration reached 0.4%, an initial gel network was formed, though the resulting gel remained soft and fragile with limited self‐supporting capacity. As the *κ*‐carrageenan content increased, the outer firmness of the EWP/Car gels improved, and the gel structure became more consolidated. However, when the carrageenan concentration exceeded 0.6%, the internal structure appeared rough, with the formation of hollow regions in the center. At 1.0% carrageenan addition, pronounced phase separation was observed. These results indicate that moderate addition of *κ*‐carrageenan significantly enhances the structural integrity and formability of thermally induced EWP gels, while excessive carrageenan levels lead to phase separation and a looser gel network.

### 3.2 WHC and LF‐NMR Determination

**FIGURE 2:**
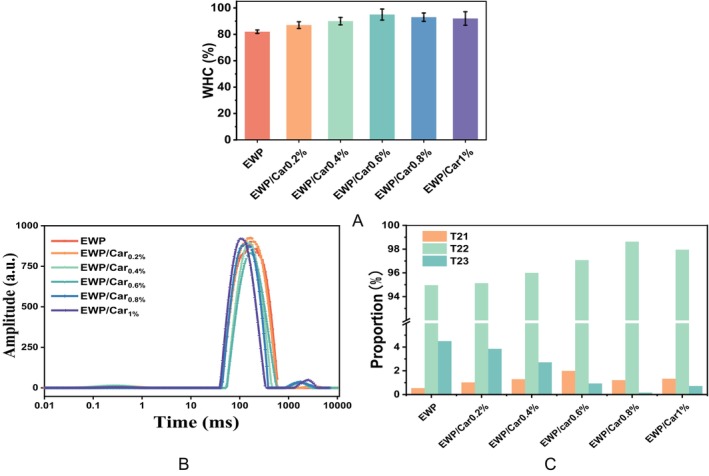
WHC (A), T_2_ relaxation time distributions (B), and peak area proportion of T_21_, T_22_, and T_23_ components (C) of different gels.

To further assess the effect of *κ*‐carrageenan on water distribution and retention, both water‐holding capacity (WHC) and low‐field nuclear magnetic resonance (LF‐NMR) measurements were performed. WHC is the ability of a protein gel or matrix to retain water within its structure. This capacity is influenced by the hydrogen bonds, hydrophobic interactions, and electrostatic interactions within the protein molecules, which affect their ability to interact with water molecules. Additionally, interactions between proteins and other components also play a critical role in determining the WHC (Babaei et al. 2019; Jin et al. 2021). The WHC of the heat‐induced WHP/Car gels is shown in Figure 2A. The WHC of EWP/Car gels was statistically superior to that of the EWP gels alone (*p* < 0.05). Furthermore, with the enhancement of *κ*‐carrageenan concentration, the WHC of the gel further improves (*p* < 0.05). The WHC of all composite gels remained above 90%. We speculate that *κ*‐carrageenan plays a synergistic role in the egg white system, facilitating tighter integration of the existing complexes, such as low‐density lipoproteins (LDL), with free proteins, thereby enhancing the water‐holding capacity of the egg gel. Additionally, *κ*‐carrageenan may also provide extra binding sites (—OH) for water molecules, forming a network that helps retain moisture and prevent water loss (Dongling et al. 2023).

Low‐field NMR can be a powerful tool for analyzing the binding state of water molecules. Measuring the T_2_ relaxation time provides an accurate indicator of water molecule mobility and freedom (Zhang, Mei, et al. 2024). As shown in Figure 2B, the relaxation time distribution of the composite gel contains three distinct relaxation time peaks at 0–10 ms (T_21_), 10–600 ms (T_22_), and 1000–5000 ms (T_23_), which represent bound water, immobilized water, and free water, respectively. The T_2_ relaxation time of the EWP/Car composite gel exhibits a significant reduction, with the shortest T_2_ observed at Car concentrations of 0.6% and 0.8%. The T_2_ relaxation time was directly proportional to the mobility of water molecules. A shorter T_2_ relaxation time signifies diminished mobility of water molecules. The diminished mobility of water molecules implies restricted freedom within the gel matrix and intensified protein interactions (Xin et al. 2023).

From Figure 2C, it can be observed that the proportion of bound water (T_21_) peak increases with the addition of *κ*‐carrageenan. The immobilized water (T_22_) peak constitutes the largest percentage, which decreased and then increased overall with the addition of *κ*‐carrageenan, and the immobilized water peak area of EWP/Car_0.8%_ gel had the largest percentage. Furthermore, the presence of free water significantly decreases upon the addition of *κ*‐carrageenan. These observations suggest that *κ*‐carrageenan promotes the transformation of free water into immobilized and bound water within the gel matrix, resulting in a more efficient binding between water molecules and proteins. This leads to a tighter and more stable gel structure (Dongling et al. 2023).

### 3.3 TPA

**FIGURE 3:**
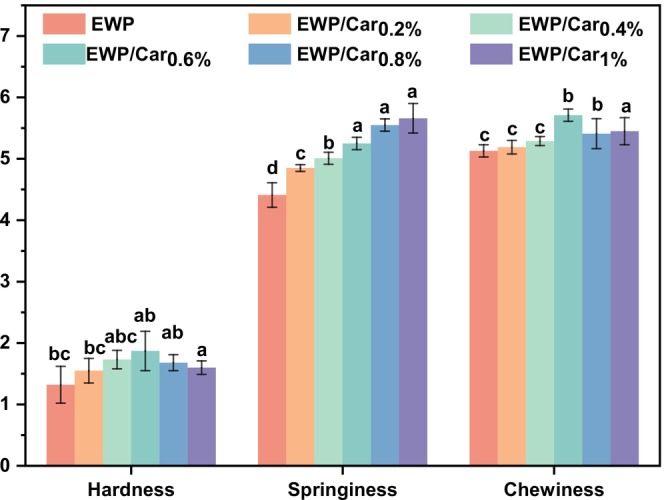
TPA of samples. All data represent the mean of triplicates. Different letters represent significant differences (*p* < 0.05).

Gel texture analysis is one of the indicators for the mechanical properties of gel and is key to determining their suitability for use in foodstuffs (Cubides et al. 2019). The increase in *κ*‐carrageenan concentration enhances the interactions between *κ*‐carrageenan and egg white protein molecules, including hydrogen bonding and hydrophobic forces, forming a tight and stable network structure (Hu et al. 2021). As shown in Figure 3, the hardness, elasticity, and chewiness of the EWP/Gar gels were significantly enhanced with the addition of *κ*‐carrageenan.

Among them, the EWP/Car_0.6%_ gel exhibited the highest hardness and chewiness. This can be attributed to the formation of a denser protein gel, which provides greater gel strength and enables the structure to withstand external compression. In contrast, a decrease in hardness and chewiness was observed in the EWP/Car_0.8%_ and EWP/Car_1%_ gels. When analyzed alongside Figure 6, this reduction may result from excessive *κ*‐carrageenan concentration, leading to over‐crosslinking or aggregation, which disrupts network uniformity and compromises the overall gel stability (Babaei et al. 2018). This finding aligns with our CLSM observations, where the addition of *κ*‐carrageenan facilitated the formation of a more compact network structure.

### 3.4 Measurements of Rheological Properties

**FIGURE 4:**
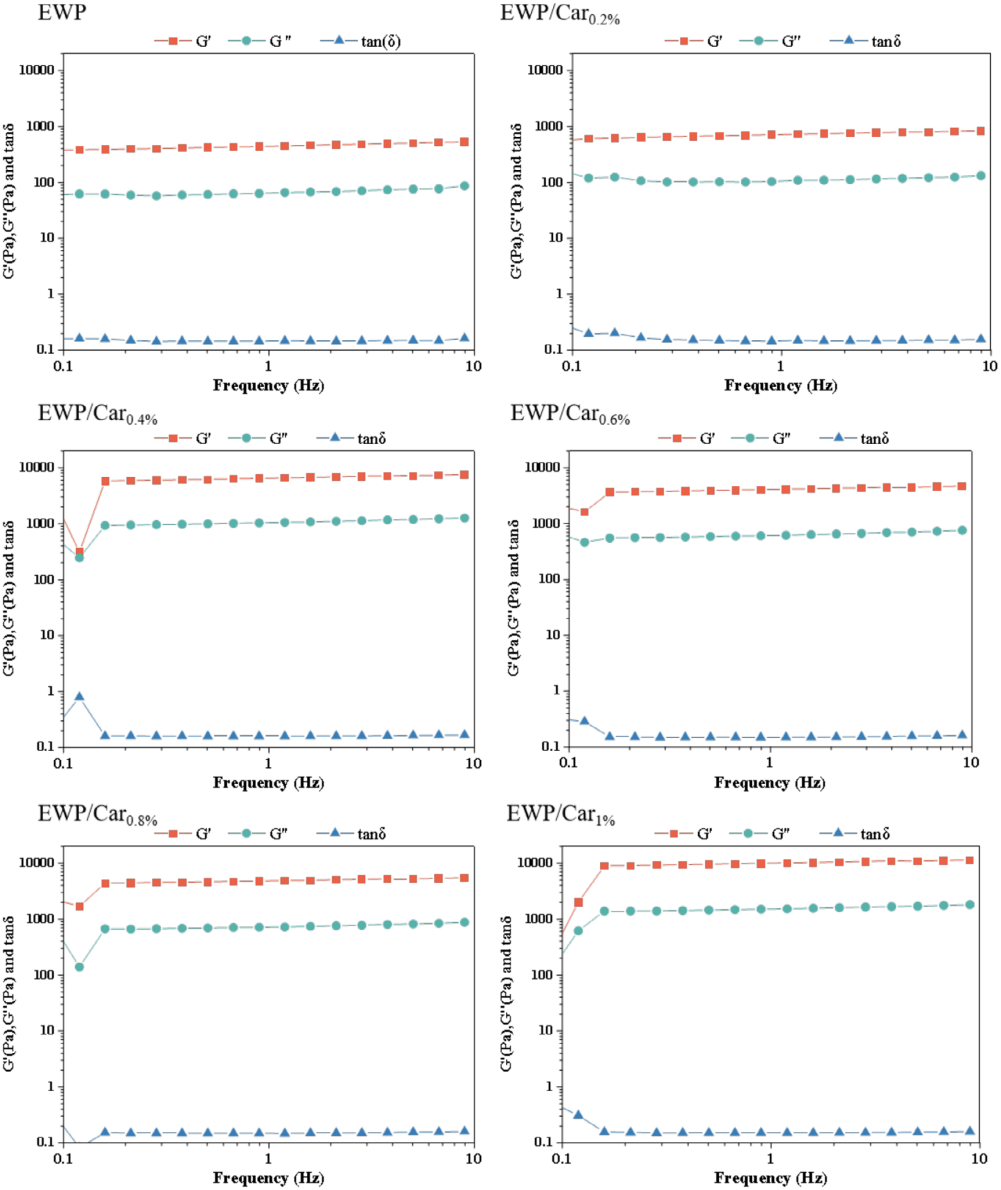
G', G″, and tan δ as a function of frequency for EWP and different concentrations of EWP/Car gels.

As shown in Figure 4, the storage modulus (G) of all six samples consistently exceeded the loss modulus (G″) across the entire frequency range. Previous studies have indicated that G″ being greater than G″ and both moduli running parallel is a typical characteristic of gel systems. Thus, all samples exhibited gel‐like behavior. The tanδ values remained below 1, suggesting that elastic behavior dominated in all gels. Notably, G' gradually increased from EWP to EWP/Car_0.8%_, indicating that the addition of *κ*‐carrageenan enhanced network formation. EWP/Car_0.8%_ exhibited the highest G' and the lowest tanδ, implying an optimal interaction between egg white proteins and *κ*‐carrageenan, resulting in a more compact gel matrix. In contrast, EWP/Car_1%_ showed a slight decrease in G' and an increase in tanδ, which may be attributed to phase separation or carrageenan self‐aggregation at higher concentrations.

### 3.5 Microrheology Analysis

Micro‐rheology offers a non‐invasive method for characterizing the viscoelastic behavior of gels at the microscale. In this study, we employed diffusing wave spectroscopy to monitor the Brownian motion of tracer particles within the gel matrix (Zhao et al. 2023). This method characterizes the rheology of the sample by monitoring fluctuations in the intensity of scattered light over time (Jin et al. 2021).

Among the parameters derived from DWS, the transport mean free path (L*) serves as an indicator of photon penetration depth. The transport mean free path (L*) refers to the depth to which light can penetrate into a sample. L* depends on the concentration and size of particles within the sample, as well as the molecular structural state (extended or coiled) of the sample. When larger particles or more regular interfaces are present in the medium, photons are less likely to scatter. Conversely, when the particles in the medium are smaller or the interfaces are irregular, photons are more likely to scatter, thereby increasing the distance photons can travel within the medium (Zhang et al. 2017).

**FIGURE 5:**
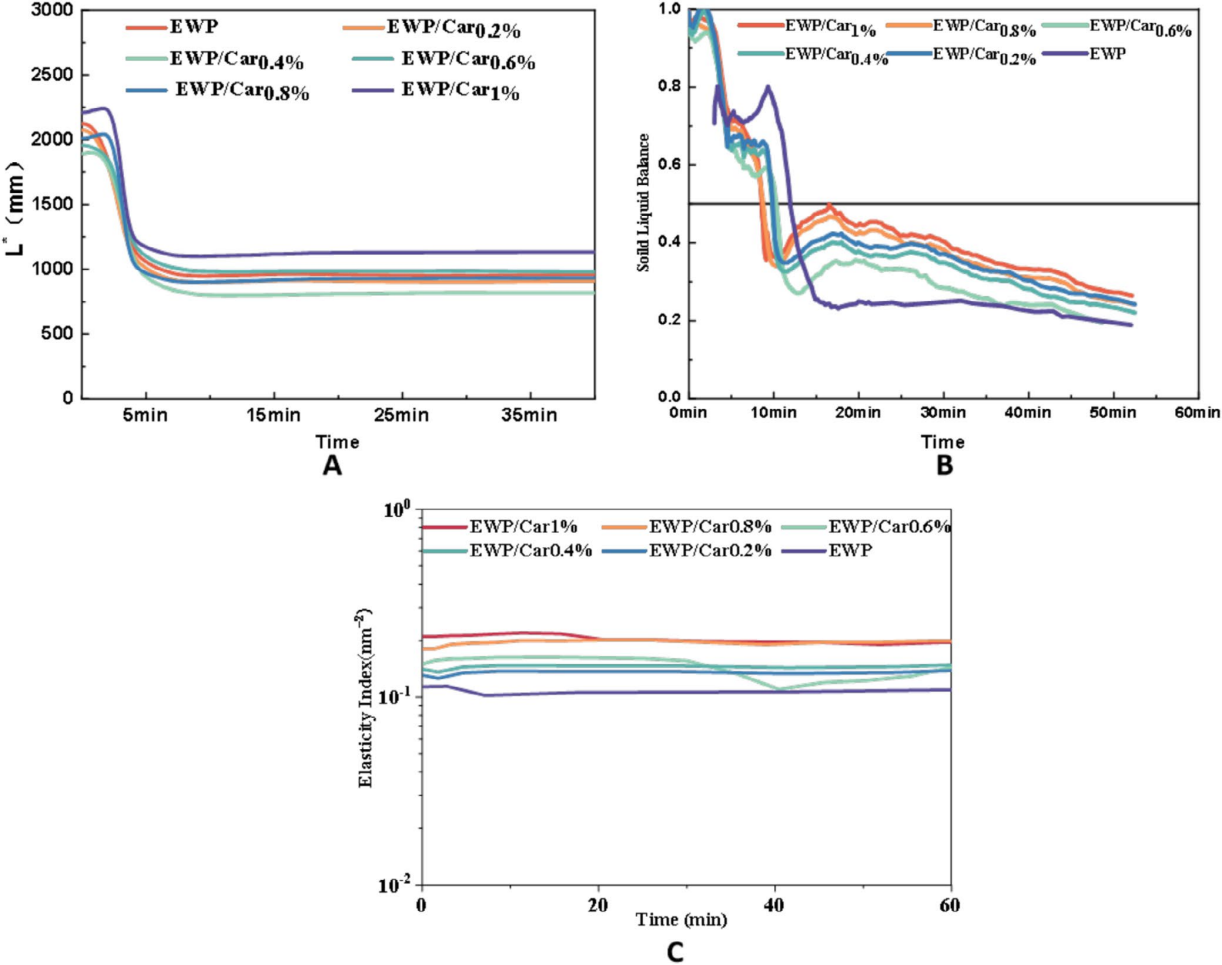
Time‐dependent microrheological properties of EWP/Car composite gels: (A) L*, (B) SLB, and (C) EI.

As shown in Figure 5A, the L* values of EWP and EWP/Car gels show a biphasic decay: a rapid decline within 0–5 min (Phase I), followed by a slower reduction until stabilization (Phase II). For egg white protein molecules, the extent of molecular chain extension affects the L* values. More extended protein molecules have larger L* values because their small molecular framework makes light scattering difficult. Conversely, tightly coiled protein molecules increase scattering within the medium, leading to a rapid decrease in L* values. As the gel network forms and the protein structure stabilizes, the L* values gradually reach equilibrium. Additionally, the coiling of *κ*‐carrageenan also contributes to the L* values.

In this experiment, we also observed that the L* values of EWP/Car gels are lower compared to EWP gels. At the high temperature of 90°C, *κ‐*carrageenan molecules should be in an extended state, which contributes to the L* values, but this contribution is likely minimal. Therefore, we hypothesize that *κ*‐carrageenan can interact with EWP, forming a cross‐linked network, resulting in a tighter structure and increased gel stability. As the concentration of *κ*‐carrageenan increases, the cross‐linking effect is enhanced, making the gel structure more compact, leading to a decrease in L* values. These changes indicate that EWP and Car combine to form a tightly coiled structure. The steric hindrance between molecules is smaller, and the molecules are easier to get closer to form covalent bonds between molecules to form a gel. When the carrageenan concentration exceeded 0.6%, a slight increase in L* was observed, indicating possible network heterogeneity due to phase separation or over‐gelation effects. These findings are consistent with previous structural observations and further validate the densification effect of *κ*‐carrageenan at moderate concentrations (Salvia‐Trujillo et al. 2015).

The solid–liquid balance value (SLB) is closely related to the viscoelasticity of the sample, being directly proportional to these properties. It is commonly used to analyze the transition from liquid to semi‐solid or solid state under heating conditions (Badar et al. 2023). The SLB value at the solid–liquid balance point is 0.5. When 0.5 < SLB < 1, the sample primarily exhibits liquid characteristics. When 0 < SLB < 0.5, the sample primarily exhibits solid characteristics, such as gel formation (Wang et al. 2018). As shown in Figure 5B, the SLB for all samples after heating for a period of time was below 0.5, indicating that the gels were highly viscous and elastic. Initially, all gels displayed higher SLB values; however, these decreased over time and eventually stabilized. This trend is attributed to the exposure of hydrophobic groups in the EWP upon heating, leading to hydrophobic aggregation and the formation of a three‐dimensional network. In addition, we observed that the gel formed faster after adding *κ*‐carrageenan; that is, the addition of *κ*‐carrageenan will affect the gelation time.

The elasticity index (EI) can analyze the elasticity and structure of a gel. A higher EI value indicates a denser intermolecular network and reduced free space for particle movement, resulting in increased elasticity and stronger resistance to external forces (Xu, Jia, et al. 2023).

As depicted in Figure 5C, the EWP gel exhibited the lowest EI value, indicating minimal elasticity. Increasing the *κ*‐carrageenan concentration (from 0.4% to 1%) leads to enhanced elasticity in the EWP. This increase in elasticity is due to the interaction between EWP and *κ‐*carrageenan, which promotes tighter coiling of the protein molecules, facilitating gel formation. Zhang et al.'s study found that a mixture of β‐lactoglobulin and *κ‐*carrageenan forms a network structure when heated to 90°C, supporting the observations of enhanced network formation through similar interactions (Zhang and Foegeding 2003).

### 3.6 Microstructure of Gels

**FIGURE 6:**
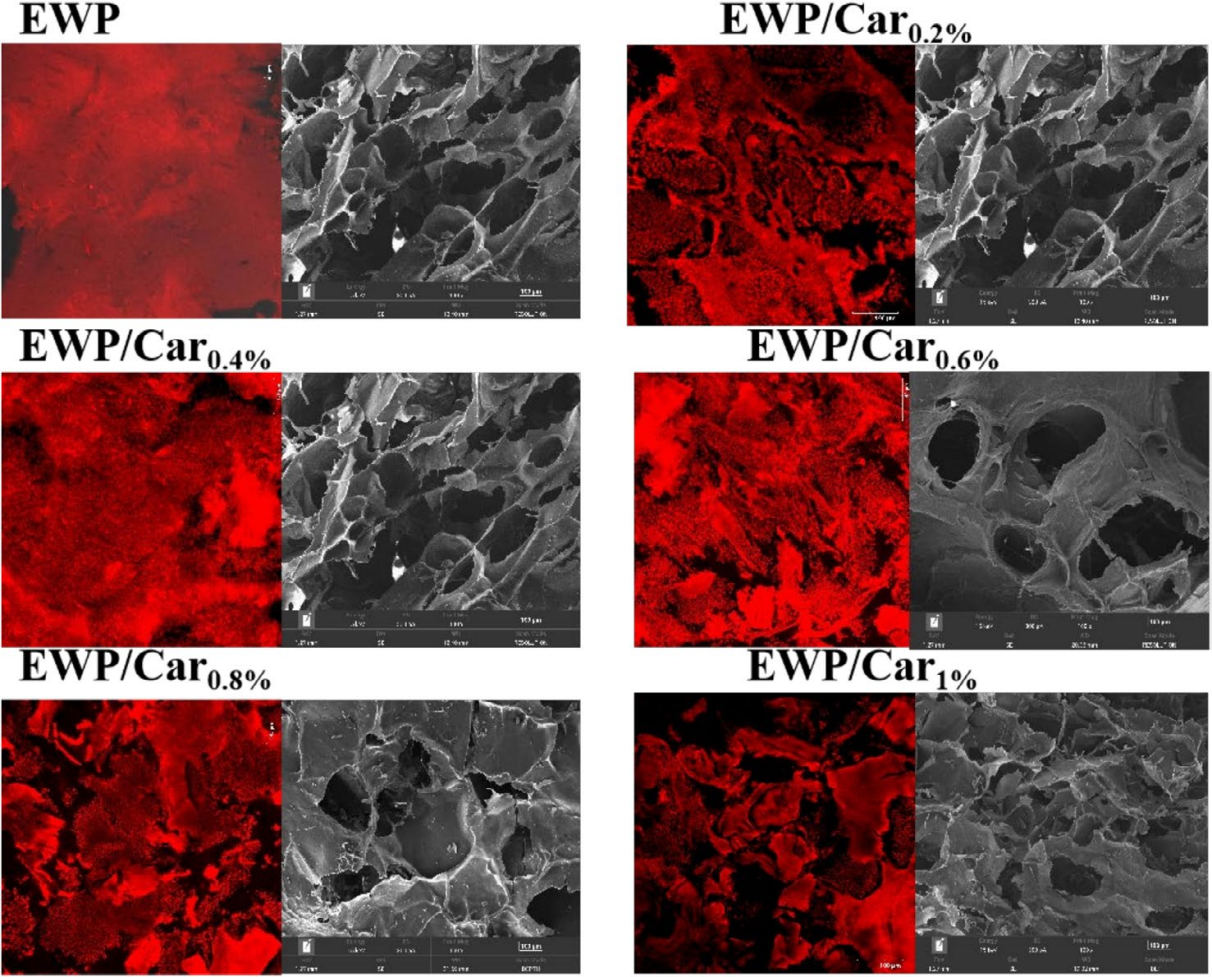
CLSM (left) and SEM (right) images of thermally induced EWP/Car composite gels prepared with varying *κ*‐carrageenan concentrations.

To further elucidate the effects of *κ*‐carrageenan on the gelation behavior and spatial organization of EWP, both CLSM and SEM were employed to visualize the microstructure of the composite gels (Figure 6).

The CLSM image of the EWP gel shows sparse and unevenly distributed red regions, indicating a loose gel network with insufficient strength and connectivity. Upon the addition of *κ*‐carrageenan, the red regions in the CLSM images become denser, suggesting that *κ*‐carrageenan promotes protein–protein crosslinking and the formation of a tighter gel network. Among the samples, EWP/Car_0.6%_ exhibits the most uniform and compact red regions, indicating that the composite network formed between EWP and *κ*‐carrageenan at this concentration is the most stable, with optimal structural integrity, elasticity, and flexibility. In contrast, the EWP/Car_0.8%_ gel shows some aggregation, likely due to excessive *κ‐*carrageenan concentration, leading to over‐crosslinking and uneven network formation. The gel appeared to form an obvious two‐phase structure (protein‐enriched and carrageenan‐enriched regions) after the addition of carrageenan of more than 0.6%, indicating that the high concentration of carrageenan led to a decrease in the compatibility of proteins and polysaccharides. In addition, the dark zone enlarged with increasing amounts of *κ*‐carrageenan, which disrupted the continuity of the protein phase and increased the density of the gel network structure. The increase in the dark zone can be attributed to *κ*‐carrageenan‐induced structural coarsening, which may be due to enhanced electrostatic interactions and hydrogen bonding. In addition, carrageenan, as an anionic polysaccharide, may bind to positively charged proteins (e.g., lysozyme) via electrostatic interactions. At low concentrations, this interaction may promote cross‐linking and the formation of denser networks. However, at high concentrations, too much carrageenan may lead to charge shielding or competition for binding, allowing the protein interactions to weaken and turn into phase‐separated structures. Dongling et al. observed analogous changes in the composite gel of soy protein isolate/λ‐carrageenan. The addition of carrageenan to protein systems in the appropriate range increased their network density, suggesting that the hard backbone and high sulfate content of *κ*‐carrageenan play a key role in the reinforcement of the gel matrix of different protein systems. Compared with soy protein isolate, egg white protein has higher surface hydrophobicity and more active thiol groups, which may lead to stronger non‐covalent interactions and a denser gel network (Dongling et al. 2023).

SEM observations further supported these findings (Figure 6). The EWP gel displayed a rough surface with loosely packed structures and irregular, large pores. With 0.2% and 0.4% *κ*‐carrageenan, a porous but increasingly interconnected network began to form. Notably, the EWP/Car_0.6%_ sample exhibited the most compact, uniform, and finely structured morphology, characterized by small, evenly distributed pores and a continuous gel matrix. However, further increasing the *κ*‐carrageenan concentration to 0.8% and 1.0% led to structural deterioration, as evidenced by the emergence of larger, irregular cavities, signs of gel collapse, and even lamellar deposits on the surface—likely due to excessive carrageenan causing phase separation or network oversaturation.

### 3.7 The Secondary Structure

**FIGURE 7:**
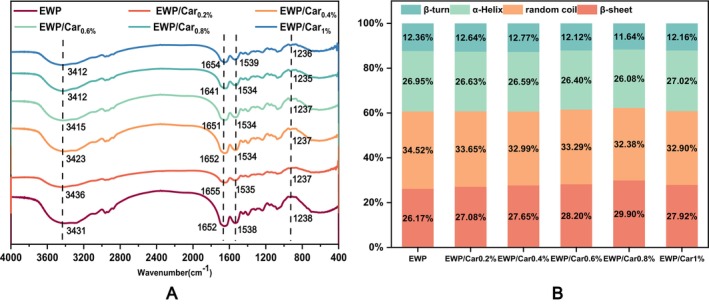
Secondary structure changes of different gels.

As shown in Figure 7A, the FTIR spectra of the four gel samples exhibit a high degree of similarity, and no new absorption peaks are observed after the addition of Car. This suggests that the main interactions between EWP and Car molecules are non‐covalent in nature. The absorption peak in the range of 3200–3600 cm^−1^ corresponds to the stretching vibration of —OH groups. Upon adding *κ*‐carrageenan, this peak shifts to a lower wavenumber, likely due to hydrogen bonding interactions between hydroxyl groups in the protein and water molecules, where a shift towards lower wavenumbers indicates stronger interactions with water (Sinthusamran et al. 2017).

The wavenumber range of 1600–1700 cm^−1^ is characteristic of the secondary structure of proteins. To further analyze the impact of *κ*‐carrageenan addition on EWP secondary structure during the thermal gelation process, second derivative and Gaussian curve fitting of the Amide I band (1600–1700 cm^−1^) in the FTIR spectra were performed to determine the relative content of each secondary structure. The Amide I band is primarily associated with C=O stretching and CN stretching vibrations, including β‐sheets (1600–1637 cm^−1^), random coils (1637–1645 cm^−1^), α‐helices (1645–1665 cm^−1^), and β‐turns (1665–1700 cm^−1^). According to Figure 7B, the relative content of the four secondary structures in the protein gel samples shows minimal variation, with EWP/Car gels showing almost no difference compared to EWP gels alone. *κ*‐Carrageenan, as a negatively charged polysaccharide, can interact with protein molecules through electrostatic interactions. This interaction is unlikely to cause significant changes in the secondary structure of proteins but may contribute to stabilizing the existing secondary structure, preventing excessive unfolding. Moreover, such aggregation behavior is more likely to manifest in the macroscopic properties of the gel (e.g., rheology, texture), rather than being directly reflected in notable changes in the secondary structure (Chen et al. 2020).

### 3.8 SDS‐Page

**FIGURE 8:**
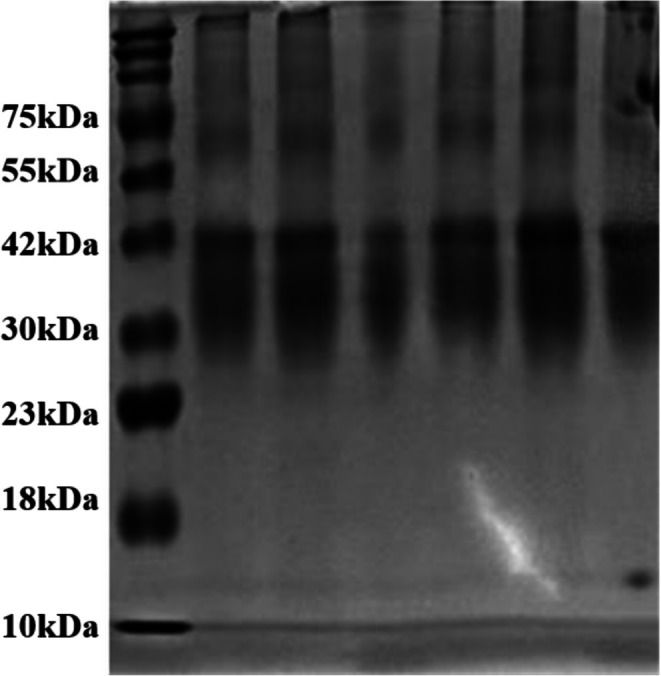
SDS‐PAGE profile of the different gels. From left to right, EWP, EWP/Car_0.2%_, EWP/Car_0.4%_, EWP/Car_0.6%_, EWP/Car_0_._8%_, and EWP/Car_1%_.

SDS‐PAGE is a widely used technique to analyze the molecular weight of proteins. Previous studies have identified lysozyme, ovalbumin (OVA), and ovotransferrin (OT) as the major protein components in EWP, distributed at 14, 45, and 76 kDa, respectively (Wang et al. 2020). Heat treatment at 90°C for 30 min was applied to induce gelation in the EWP/Car system. This thermal process likely caused irreversible denaturation and aggregation of the major egg white proteins. As shown in Figure 8, SDS‐PAGE analysis revealed distinct bands at approximately 45 and 75 kDa, corresponding to ovalbumin and ovotransferrin, respectively. The lysozyme band was absent across all samples, which may be attributed to its relatively low abundance in egg white and its thermal instability beyond 77°C, leading to denaturation and potential aggregation beyond detection (Kudre et al. 2018). Notably, there were no significant changes in the bands between EWP/Car and EWP gel, indicating that the interaction between EWP and Car primarily involves non‐covalent interactions such as electrostatic forces and hydrogen bonding. These results are consistent with the FTIR findings and provide further support for the hypothesis that *κ*‐carrageenan enhances gel strength primarily through physical network reinforcement rather than chemical modification of protein structures.

### 3.9 Intermolecular Force Analysis

Intermolecular forces are crucial in the formation of protein gels (Cao and Mezzenga 2020). To better understand the gelation mechanisms of EWP and Car, it is essential to explore both covalent (disulfide bonds) and non‐covalent interactions (ionic bonds, hydrogen bonds, and hydrophobic interactions) that occur during the gelation process (Wang et al. 2020). Under high‐temperature conditions, the increased thermal motion of water molecules leads to the aggregation of hydrophobic molecules, forming hydrophobic cores. As proteins unfold, their hydrophobic residues become exposed to the aqueous environment, leading to an increase in hydrophobic interactions between neighboring protein molecules (Liu et al. 2023). The disulfide bonds are covalent bonds formed through oxidation–reduction reactions between cysteine residues. High temperature enhances these oxidation reactions, promoting the formation of disulfide bonds, which are integral to stabilizing the protein structure within the gel (Xu, Gao, et al. 2023). Hydrogen bonds and ionic bonds are common intermolecular interactions, but their strength and stability may decrease under high‐temperature conditions.

**FIGURE 9:**
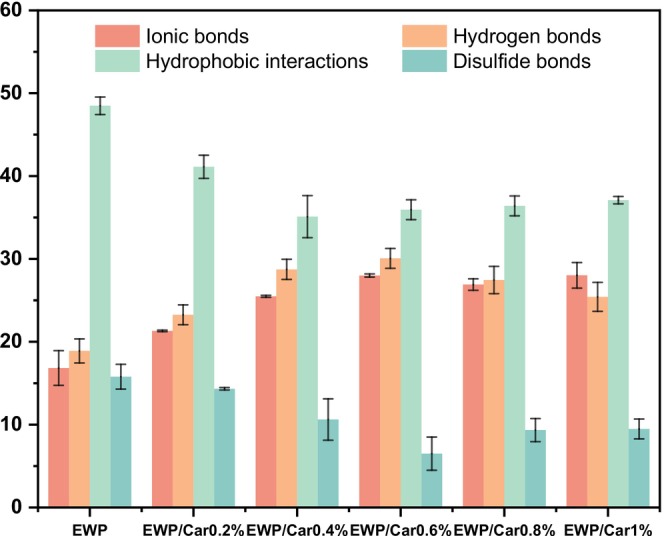
Molecular force changes of different gels.

As shown in Figure 9, hydrophobic interaction is the main driving force of EWP gel, in the range of 0%–0.6% carrageenan addition. With the increase of carrageenan addition, the hydrophobic force and disulfide bonding weakened, and ionic bonding and hydrogen bonding increased accordingly. Carrageenan and EWP form a complex wrapping the hydrophobic region of the protein through electrostatic attraction and covering the sulfhydryl group, which enhances ionic bonding and at the same time inhibits the hydrophobic interactions and disulfide bonding. The formation of disulfide bonds was inhibited. At the same time, the hydroxyl groups of carrageenan formed additional hydrogen bonds with the polar groups of the protein, resulting in a denser gel network in EWP/carrageenan. In contrast, excess carrageenan (> 6%) formed a separate polysaccharide network, which phase separated from the protein network, resulting in a loose structure. Combined with the CLSM images (Figure 6), it can be seen that with carrageenan additions > 6%, the carrageenan phase separated from the protein, and the gel properties decreased.

### 3.10 SH Group Content of Gels

The presence of sulfhydryl groups can reflect the stability of the secondary and tertiary structures of proteins. Oxidized sulfhydryl groups form disulfide bonds that play an important role in protein folding and stability. *κ*‐Carrageenan can convert sulfhydryl functional groups in proteins into their oxidized form and react with other sulfhydryl functional groups, resulting in the formation of new intermolecular disulfide bridges and promoting the formation of a gel network (He et al. 2023).

**FIGURE 10:**
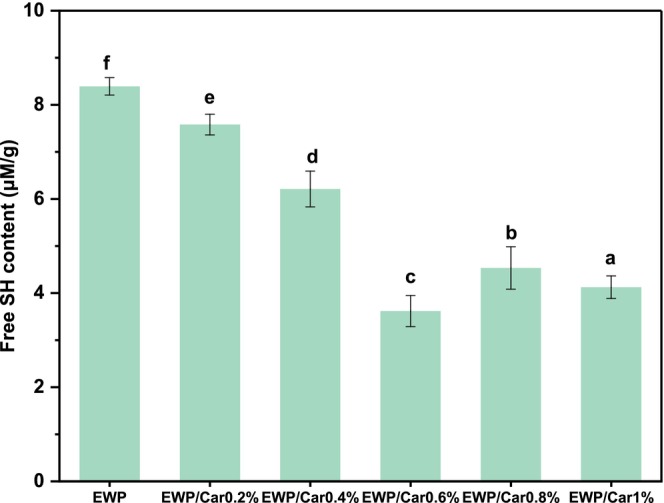
Effect of the free sulfhydryl groups content of different gels. Different letters represent significant differences (*p* < 0.05).

As depicted in Figure 10, the sulfhydryl content in the gels varies with the amount of *κ*‐carrageenan added. Within the range of 0%–0.6% of *κ*‐carrageenan addition, the content of free thiols significantly decreases. On the one hand, it may be the oxidation of sulfhydryl groups or other forms of chemical modification that lead to the reduction of sulfhydryl content. Additionally, functional groups, such as hydroxyl groups in *κ*‐carrageenan, may competitively bind with free sulfhydryl groups to form sulfhydryl–carrageenan complexes, further reducing the availability of free sulfhydryl groups (Ma et al. 2022). This observation is corroborated by the data shown in Figure 9; the EWP/Car_0.6%_ gel has the highest content of disulfide bonds, while the gel containing EWP/Car_0.4%_ and the EWP gel show a slight increase in disulfide bond content. Therefore, it can be inferred that *κ*‐carrageenan addition leads to a decrease in free sulfhydryl content.

Interestingly, Figure 10 also shows a slight increase in sulfhydryl content in the EWP/Car_0.8%_ and EWP/Car_1%_ gel. This suggests that with further increases in *κ*‐carrageenan concentration, the interactions between molecules may be enhanced, thereby reducing the competitive binding of *κ*‐carrageenan to free sulfhydryl groups (Zhang et al. 2019). As a result, *κ*‐carrageenan molecules may be more inclined to dissociate, allowing more free sulfhydryl groups to remain in their unbound state.

## 4 Conclusion

This study investigated the effects of *κ*‐carrageenan addition on the gelation properties of EWP gels, focusing on texture, water distribution, molecular interactions, and microstructure. At an optimal level of 0.6%, *κ*‐carrageenan significantly improved gel hardness, elasticity, and WHC, as evidenced by TPA, LF‐NMR, and rheological analysis. CLSM and SEM confirmed the formation of a denser and more uniform network, while FTIR and SDS‐PAGE indicated that these improvements were driven by non‐covalent interactions without altering protein secondary structures. These findings provide valuable insights into protein–polysaccharide gel systems and offer practical strategies for designing high‐moisture, protein‐rich food products. Specifically, EWP/Car_0.6%_ gels show potential for applications in egg‐based cold dishes, ready‐to‐eat protein snacks, or 3D‐printed nutrition gels. The enhanced gel stability under thermal processing also suggests its suitability in forming emulsified or aerated food matrices. Future studies should further investigate the influence of processing conditions such as pH, ionic strength, and storage on gel stability and sensory attributes, to advance the industrial application of EWP/*κ*‐carrageenan composite gels.

## Author Contributions

**Yuping Wei:** conceptualization (equal), data curation (lead), formal analysis (equal), software (lead), writing – review and editing (lead). **Weicong Lin:** methodology (equal), software (equal), visualization (equal). **Shaomin Lin:** methodology (equal), software (equal), validation (equal), visualization (equal). **Ying Nie:** funding acquisition (supporting), investigation (equal), resources (supporting). **Xianghui Zou:** formal analysis (equal), investigation (supporting), supervision (equal). **Yuzhong Zheng:** funding acquisition (supporting), supervision (equal). **Biansheng Li:** conceptualization (equal), supervision (equal). **Yuwei He:** resources (equal), software (equal). **Yisheng Huang:** conceptualization (equal), data curation (equal), methodology (equal), supervision (equal), writing – review and editing (supporting). **Yongping Huang:** conceptualization (equal), funding acquisition (lead), project administration (equal), supervision (equal), writing – review and editing (supporting).

## Conflicts of Interest

The authors declare no conflicts of interest.

## Data Availability

The data that support the findings of this study are available from the corresponding author upon reasonable request.
